# An Aberrant Case of Sacrococcygeal Teratoma in a Female Infant Born of Twin Pregnancy

**DOI:** 10.7759/cureus.37288

**Published:** 2023-04-08

**Authors:** Gajanan K Wattamwar, Avinash Dhok, Suresh Phatak, Kajal Mitra, Suruchi Dhawan, Swaragandha S Jadhav

**Affiliations:** 1 Department of Radiology, NKP (Narendra Kumar Prasadrao) Salve Institute of Medical Sciences, Lata Mangeshkar Hospital, Nagpur, IND

**Keywords:** case report, infantogram, sacrococcygeal mass, teratoma in twins, sacrococcygeal teratoma

## Abstract

Sacrococcygeal teratoma (SCT) is an uncommon infantile tumor. It has a female preponderance with malignant variants being more common in males. These usually manifest as palpable masses over the sacral region in infancy which may or may not be associated with neural tube defects. An initial radiological investigation is warranted to analyze the extent and components of the mass to guide an approach for surgical excision. We present a classic case of an SCT in a female infant born as a twin. This mass was evaluated radiologically by X-ray and ultrasound followed by histopathological correlation. This is a case of Altman Type-I lesion and was confirmed as a mature teratoma on histopathological examination.

## Introduction

Sacrococcygeal teratoma (SCT) is an uncommon infantile tumor. It has a female preponderance with a male-to-female ratio of 1:4. However, malignant variants of sacrococcygeal teratomas are more common in males. It has an incidence of one in 35,000-40,000 live births [1]. SCT is a congenital tumor diagnosed during gestation or early infancy. The majority of SCTs are mature teratomas, which is a subtype of germ cell tumors (GCT). Despite the histological similarities, SCT is distinguished from GCT in that the latter develops after puberty in the gonads and other extragonadal locations. Around 90% GCTs arise from gonads, particularly testis, while few cases show extragonadal locations such as the retroperitoneum, mediastinum, neck, and brain. Nonetheless, the sacral and coccyx region is the most common location of GCT formation in neonates and infants [2]. Surprisingly, the majority of SCT patients are sporadic, with no familial propensity, while a twin gestation family history is reported in 10-15% of cases. Our case is born out of a twin pregnancy and stands as an example. SCTs are typically detected at birth as lumps. Proper clinical examination plays a paramount role in establishing an initial diagnostic suspicion [2,3]. Radiological investigations play a crucial role in both prompt diagnosis as well as adequate visualization of the mass and its extent.

## Case presentation

**Figure 1:**
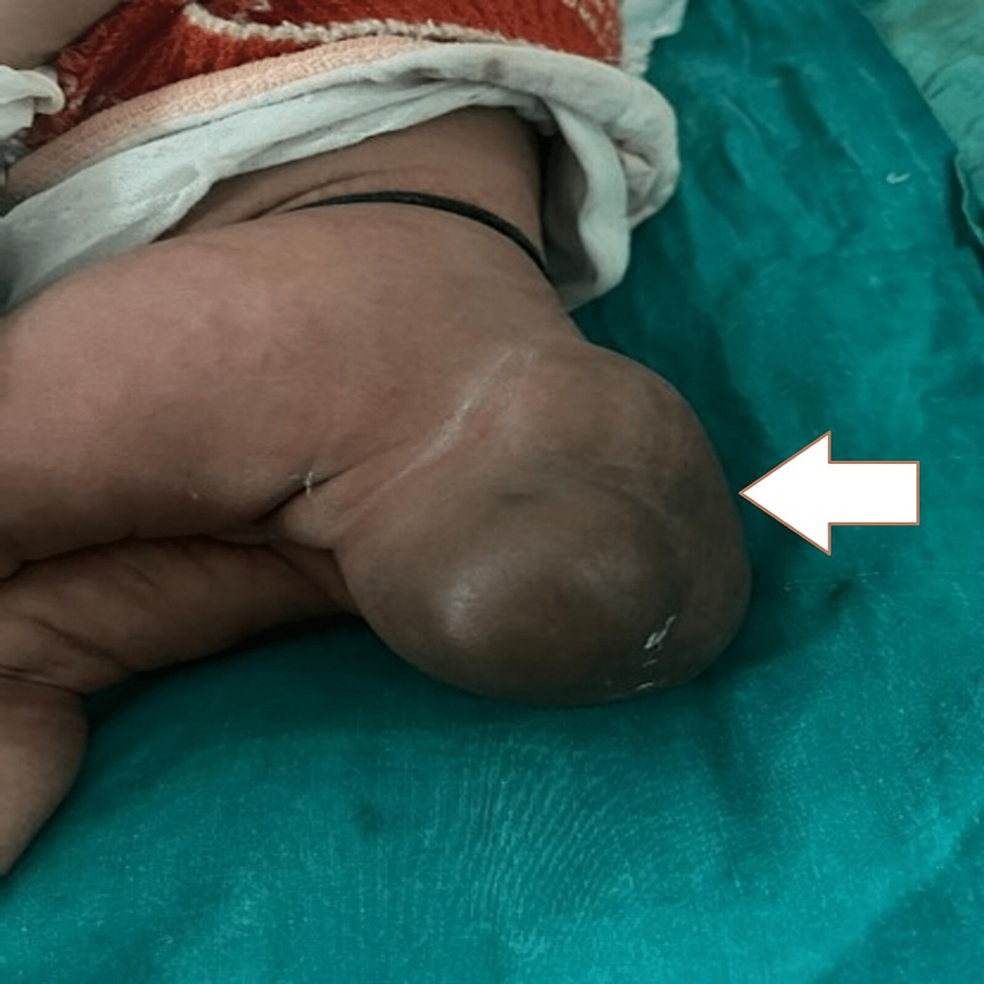
Clinical image showing the mass in the sacrococcygeal region (white arrow).

A two-month-old female born out of a twin pregnancy presented with a mass at the sacral region. The mass was present since birth and slightly increased in size over the past two months. It measured approximately 10 x 8 cm on presentation (Figure 1).

**Figure 2:**
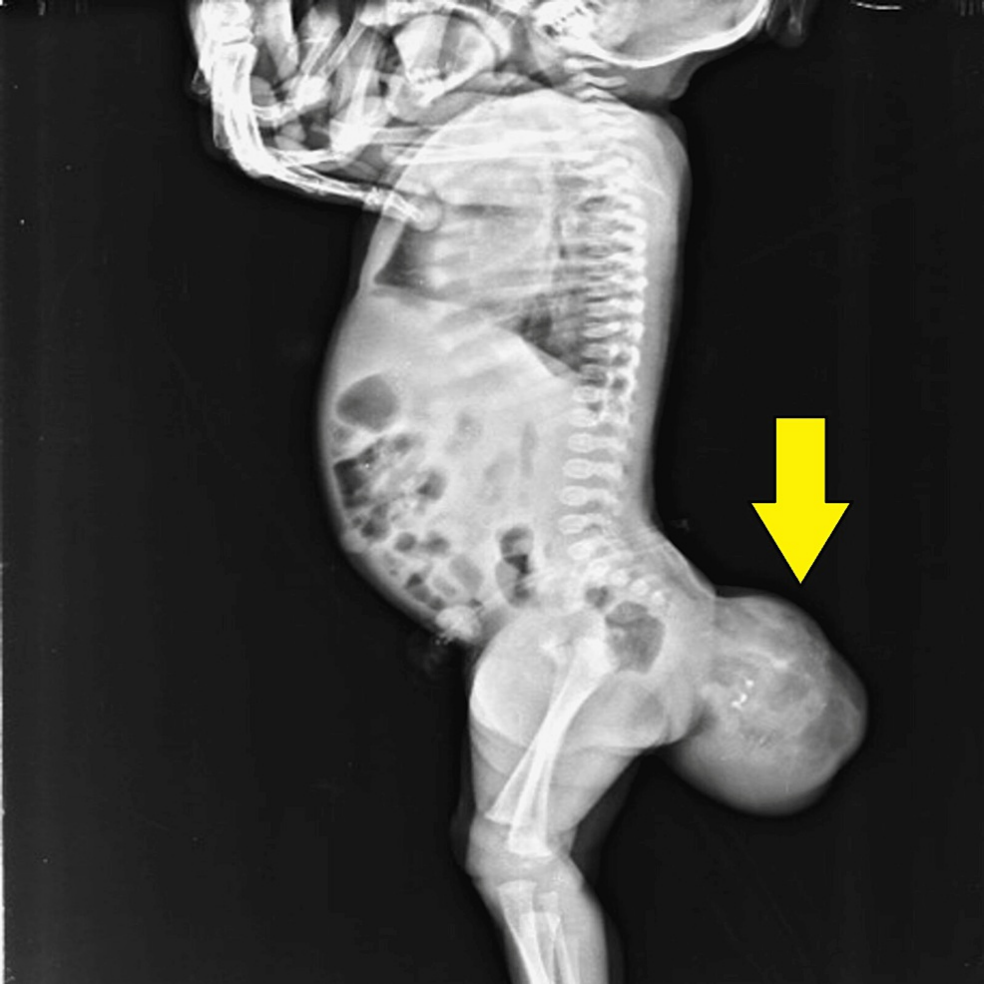
Lateral Infantogram, showing a well-defined soft tissue density mass (yellow arrow) at the sacrococcygeal region with tiny calcifications and few lucent areas representing fat. There is no underlying bony destruction.

The patient had no bowel or bladder complaints. On examination, the mass was well-defined in shape and soft in consistency. It showed irregular margins and was mobile in nature. It showed a positive fluctuation test and was displacing the anal opening anteriorly. The infant was advised X-ray infantogram including the lumbosacral spine followed by an ultrasound examination of the mass. On X-ray, a well-defined soft tissue density lesion was visible at the sacrococcygeal region, also showing tiny calcifications and few lucent areas within indicating fat. No underlying bony erosion or destruction was noted (Figure 2).

**Figure 3:**
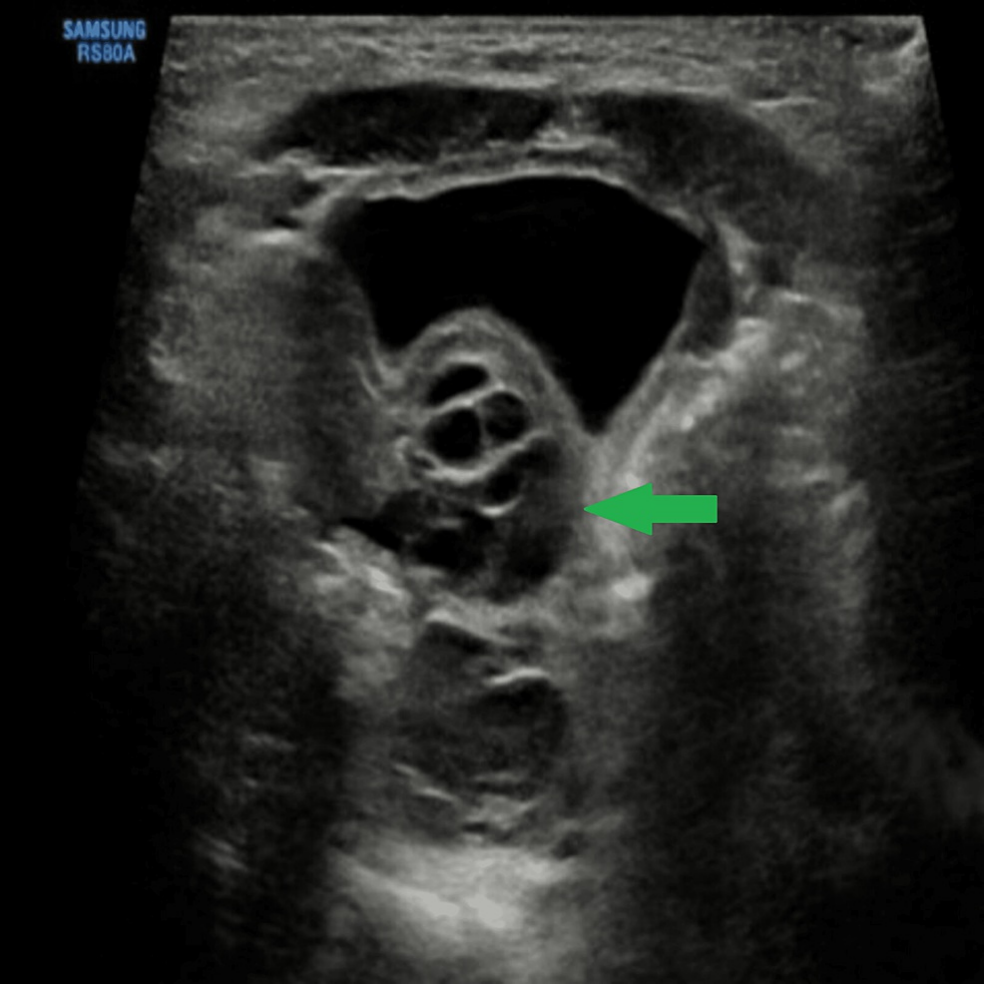
On B mode ultrasonography, multiple cystic areas (green arrow) are seen posterior to the urinary bladder showing the internal extension of the sacral lesion.

On ultrasound, a well-defined lesion with solid and cystic components was noted with fatty tissue within the solid component. Few of the cystic components were anechoic and few showed debris within. A small extension of the lesion was seen internally posterior to the urinary bladder, which was multi-cystic in appearance (Figure 3).

**Figure 4:**
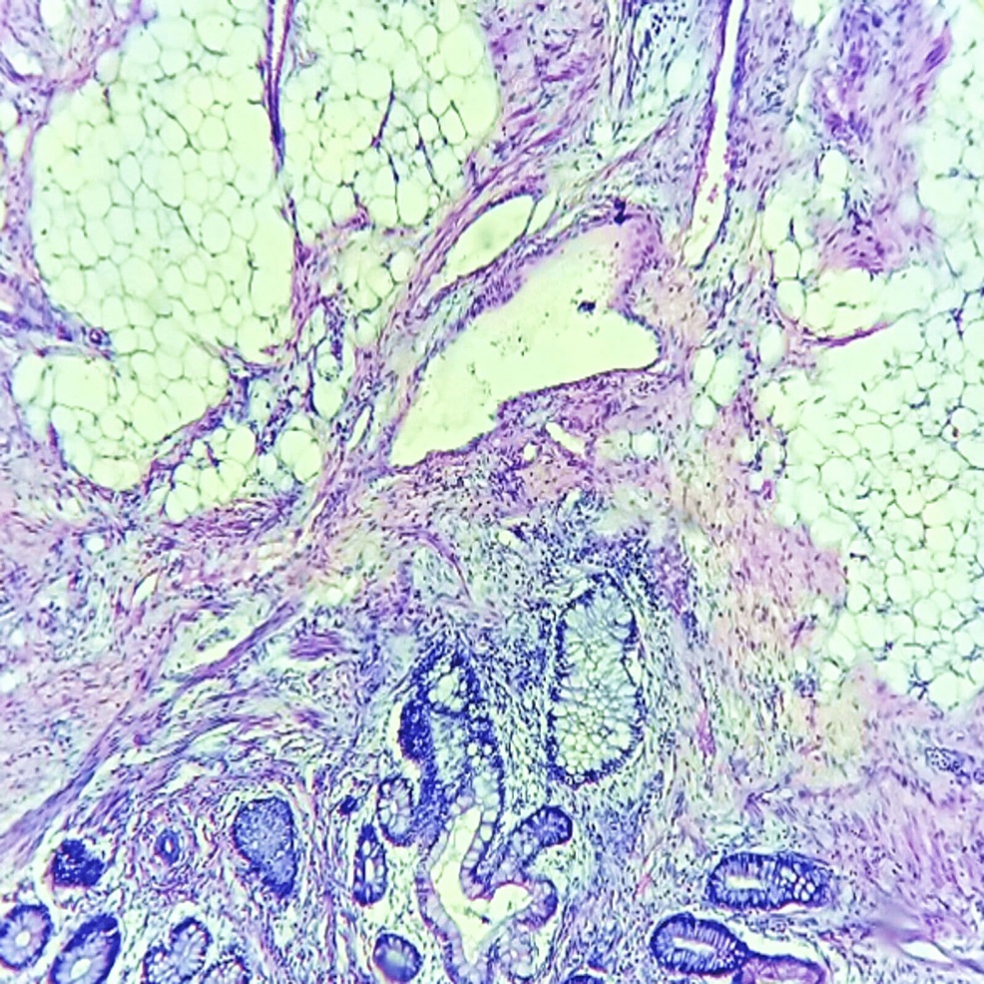
Fibromuscular tissue with adipose component and glandular tissue

The patient underwent surgical excision followed by histopathological evaluation of the specimen. On histopathology, it had areas of stratified squamous epithelium, ciliated and mucin secretion columnar epithelium, and transitional epithelium. It also had fibro adipose tissue as well as muscle bundles at places along with cartilage and adnexal structures like sebaceous glands and sweat glands (Figure 4). These features were consistent with a mature teratoma.

## Discussion

SCTs are solid tumors mainly seen in newborns. The most typical location for a teratoma in infants is the sacrococcygeal region. Adult SCTs are rare [1]. Meningocele, myelocystocele, myelomeningocele, teratoma, lipoma, hamartoma, lymphangioma, hemangioma, chordoma, and ependymoma are possible differential diagnoses of fetal sacrococcygeal masses [4]. Although, occasionally, there may be sacral dysgenesis or hemivertebrae, SCT is not primarily a neural tube abnormality and there is usually no spinal dysraphism [5].

The mainstays in the diagnosis of SCT antenatally are fetal ultrasonography and MRI. MRI describes the intrapelvic and abdominal extent of the tumors more precisely than ultrasound and it also offers more details on the compression of adjacent organs [6].

Altman et al. conducted a study and established a classification system for SCTs based on the location of tumors. In Type I, the tumor is predominantly external with a minor presacral component. Tumors having a large intrapelvic extension but a minor external component are classified as Type II. Tumors externally visible as a small lump, with the major component located internally in the pelvis and abdomen are classified as Type III. Type IV only has a presacral component with no external component. This classification helps in deciding the surgical approach and its prognosis [7].

According to Altman, our case falls in the Type I category since it has a major external component and a minor presacral component. Histopathological examination revealed that it is a mature teratoma, which was benign in nature. Although histological examination plays a crucial role in the diagnosis of SCT, a correlation of both the radiological imaging findings and the pathological examination provides a more holistic approach to diagnosis by considerably identifying the nature of teratoma as benign [6].

Standard surgical management of SCTs includes a wide excision of the tumor without rupture or spillage and a minimal amount of gluteal muscle resection with no injury to the bowel, bladder, or other pelvic organs [8].

## Conclusions

SCT is a relatively rare tumor that occurs in newborns and infants. Fortunately, with early diagnosis and proper treatment, the prognosis of SCT is generally good. The treatment approach involves complete surgical removal of the tumor, which may be challenging because of the tumor location. Altman classification is highly useful in surgical management; hence, preoperative assessment is crucial to facilitate complete excision. Overall, SCT requires a multidisciplinary approach involving pediatric surgeons, oncologists, and radiologists. With continued research and advancements in treatment, the prognosis of SCT will improve further.
